# Supplementary material for: Are carbon emissions trading and green financial instruments synergistic? -Comprehensive quantitative research based on content analysis
Source: PLoS One. 2024 Mar 7;19(3):e0298601. doi: 10.1371/journal.pone.0298601 (PMC10919601; doi:10.1371/journal.pone.0298601)
Supplement: S1 Data — (ZIP) [file pone.0298601.s001.zip › Data/地级市Z_F工作报告.zip!/─┌├╔╣┼/═¿┴╔╩╨/└┤╫╘╣1⁄2╓┌║┼í╛┬φ┐╦╩2╛▌═°í┐_2018.docx]

各位代表:

现在,我代表市人民政府向大会报告工作,请予审议,并请市政协委员和列席会议的同志们提出意见。

一、过去五年的发展变化和2017年工作回顾

过去的五年,是通辽改革发展进程中变化较大、成效明显的五年,是全市各族干部群众守望相助、同心筑梦的五年。五年来,我们认真贯彻落实党的十八大精神,以习近平新时代中国特色社会主义思想为指导,全面贯彻落实党中央决策部署和习近平总书记考察内蒙古重要讲话精神,在自治区党委、政府和市委的正确领导下,在市人大、市政协的监督支持下,统筹推进“五位一体”总体布局和协调推进“四个全面”战略布局,牢固树立和践行新发展理念,主动适应经济发展新常态,改革创新、开放转型、务实担当,完成了本届政府目标任务,各方面工作都取得了新进展、新成就。

经济发展稳中有进。坚持稳中求进工作总基调,有效克服下行压力,经济运行的稳定性、协调性明显增强。地区生产总值年均增长7%;规模以上工业增加值年均增长8.5%;限额以上固定资产投资年均增长11.7%;社会消费品零售总额年均增长10%;城乡常住居民人均可支配收入分别达到29639元和12512元,年均分别增长9.2%和9.7%。工业用电量达到372亿度,比2012年增长1倍多;货物运输总量达到1.75亿吨,比2012年增长近1倍;驻市金融机构存贷款余额均突破千亿元,城乡居民储蓄存款达到659亿元,比2012年增长近1倍。

产业转型升级步伐加快。着力转方式、调结构,制定了落实习近平总书记“五个结合”重要要求、加快转变发展方式的实施意见。依托优势建设“六大产业基地”,三次产业比调整为14:48.2:37.8,第三产业比重提高6.9个百分点。节水高产高效粮食功能区建设走在自治区前列,肉牛养殖规模和产业化水平全国领先,在自治区率先完成特色产业标准体系规划和标准制定。工业扩量提质增效,资源型产业延伸升级,战略性新兴产业规模扩大,新能源占电力总装机达到42%,发电量占比超过20%;蒙药产量占全国的50%以上。中心城区“五大物流平台”初具规模,电子商务进农村综合示范县项目全覆盖,通辽电子商务产业园被评为“全国电子商务示范基地”。完成科尔沁500公里风景大道自驾游服务系统一期建设,被命名为“全国自驾游目的地试点城市”和“中国蒙餐之都”。互联网、大数据等新业态加快成长,被评为“宽带中国示范城市”。

改革开放取得突破性进展。开展经济生态和社会事业改革试点29个,落实改革举措528项。企业减负增效工作获国务院第三次大督查通报表扬,“助保贷”模式在全区推广。制定并实施政府权责等“八张清单”,推行“双随机一公开”监管。标准化三年行动计划取得扎实进展,国家级社会管理和公共服务综合标准化试点通过验收。在自治区率先实施“二十五证合一”改革,被国家确定为东北地区民营经济发展改革示范城市。区域微型电网试点取得阶段性成效。草原确权基本完成,土地确权登记颁证国家级试点通过自治区验收。建立分级诊疗制度,公立医院全部实行药品零差率销售。积极融入“一带一路”建设,不断扩大对俄蒙开放,与蒙古国肯特省建立友好关系,举办中蒙博览会通辽国际商品交易会暨中蒙“3+3”区域合作会议,“通满欧”跨境货运班列开通运营。创新招商引资引智方式,累计到位资金3904亿元,引进高层次人才1000名。

生态环境质量明显改善。规划建设“五大生态功能区”,制定重点生态功能区产业准入负面清单,划定全域基本农田,落实最严格的水资源管理制度,完成领导干部自然资源资产离任审计试点。建立生物多样性保护示范区,罕山晋升为国家级自然保护区,孟家段水库、胡勒斯台淖尔湿地被命名国家湿地公园,奈曼宝古图、库伦银沙湾成功创建国家沙漠公园。科尔沁沙地“双千万亩”综合治理1400万亩,村屯绿化全覆盖,通道绿化3500公里,造林450万亩,被授予“全国绿化模范城市”称号。

城乡面貌发生巨大变化。坚持城乡统筹、共建共享,一批重大基础设施投入使用,通辽至鲁北等4条高速公路建成通车,新增高等级公路566公里,中心城区至旗县城关镇实现高等级公路连接,通辽至京沈高铁新民北站铁路线下工程基本完工,扎鲁特至青州±800千伏特高压直流输电工程投产运行,霍林河机场通航运营,双胜至通辽天然气管道建成使用。新一轮城市总体规划颁布实施。常住人口城镇化率达到48.7%,提高5.7个百分点。成功创建国家园林城市,辽河公园获“中国人居环境范例奖”。5个旗市成功创建“自治区园林县城”,舍伯吐镇、东风镇被评为国家级特色小镇。全力建设新农村新牧区,行政嘎查村全部通沥青(水泥)路,街巷基本全硬化,城乡面貌焕然一新。

人民群众获得感显著增强。一批惠民举措落地实施,连续两年入选“中国地级市民生发展100强”。累计整合投入资金200多亿元,扶持39.7万人稳定脱贫,贫困发生率降至3%。就业形势总体稳定,城镇新增就业10.4万人。率先在自治区实现城乡医疗保险一网化运行管理。出台失地农牧民参加养老保险政策,被国家确定为试点地区。社会救助和扶贫政策有效衔接,养老、低保、医保等保障水平和社会救助标准逐年提高。实施百姓安居工程,建设公共租赁住房3000套,改造棚户区8万户、老旧小区532个、农村牧区危土房17.8万户,近百万群众居住条件得到改善。实施农村牧区饮水安全工程929处,56万农牧民喝上安全水;实施城镇自来水水质提升工程,饮用水检测能力达到国家标准。中心城区新增公交车378台,公交出行分担率提高14个百分点、达到20%,居民出行更加便利。

各项社会事业全面进步。教育教学水平得到提升,6个旗市区通过国家义务教育均衡发展达标验收,教育信息化“三通两平台”步入自治区前列,在全区率先实现苏木乡镇、中心嘎查村幼儿园全覆盖,义务教育阶段学校“大通铺”改造和农村牧区教师周转宿舍建设全面完成。医疗卫生水平稳步提高,基层基本医疗服务全覆盖,获评全国基层蒙中医药工作先进市、“中国蒙医药之都”、全国无偿献血先进市。基层公共文化服务体系标准化、均等化建设走在自治区前列,全国贫困地区公共文化建设工作推进会在我市召开,设立“科尔沁文化政府奖”,乌兰牧骑事业取得长足发展,承办第22届全国版画展,乌力格尔等非物质文化遗产有效传承发展。举办首届市民运动会,全民健身氛围浓厚。足球改革取得新成果,新建改造足球场地300余片,取得国际国内赛事冠军11项。双拥优抚安置政策有效落实,连续四届获全国“双拥模范城”称号。民族团结进步创建活动深入开展,各民族共同繁荣进步局面持续巩固。新闻出版、人民防空、外事侨务、妇女儿童、气象地震、档案史志、科协、慈善、老龄、残疾人及关心下一代等事业取得新成效。

社会治理能力大幅提升。“平安通辽”建设扎实推进,立体化治安防控体系不断完善,城镇重要部位视频监控全覆盖。“大信访、大维稳”工作成效明显,信访维稳形势积极向好。全面推行“网格化”管理和“532”工作法,开展农村牧区社区建设试点,基层政权建设稳步推进。“六五”普法全面完成,被授予“全国法治宣传教育先进城市”。食品药品安全市创建工作通过自治区验收。严格落实安全生产责任制,强化隐患排查整治,安全生产形势总体平稳。

政府自身建设得到加强。全面建设法治政府,依法行政工作连续两年进入自治区先进盟市行列。大力推进简政放权,市本级行政许可事项精简幅度达到85%,创新监管,推行“互联网+政务服务”,更大力度利企便民。自觉接受人大法律监督、政协民主监督,积极听取民主党派、工商联、无党派人士和专家学者意见,严格执行“三重一大”制度。坚持全面从严治党,讲规矩、守纪律,扎实开展党的群众路线教育实践活动、“三严三实”专题教育、“两学一做”学习教育,严格履行党建、党风廉政建设、意识形态“一岗双责”,认真执行中央八项规定精神和自治区党委、市委配套规定,整治“四风”问题。严格落实国务院“约法三章”,党政机关和参公事业单位公务用车制度改革全面完成,政府工作作风明显转变,服务效能有新的提升。

五年的成就有目共睹、振奋人心。这些成绩的取得,是自治区党委、政府和市委正确领导的结果,是人大代表、政协委员和社会各界关心支持的结果,是各族干部群众共同努力的结果。在此,我代表市政府,向离退休老同志,向中区直单位、驻市军警部队,向一直关心支持通辽建设与发展的同志们、朋友们,致以崇高的敬意和衷心的感谢!

五年的经验弥足珍贵,概括起来就是:必须坚持正确的政治方向,坚决维护以习近平同志为核心的党中央权威和集中统一领导;必须坚持发展第一要务,把发展作为解决一切问题的基础和关键;必须坚持以人民为中心的发展思想,把人民对美好生活的向往作为奋斗目标;必须坚持生态优先、绿色发展,绝不以牺牲环境为代价换取一时的经济增长;必须坚持改革创新、开放合作,不断增创发展新优势;必须坚持守望相助、团结奋斗,守好各民族共有的精神家园。这些经验,是我们续写通辽发展新篇章的强大精神力量,必须长期坚持和不断发展。

刚刚过去的2017年是令人难忘的一年,党的十九大胜利召开,迎来自治区成立70周年。在市委的坚强领导下,我们有效应对宏观环境深刻变化,因时而动、顺势而为,完善发展思路和工作举措,解决部分经济指标虚增问题,促进了经济平稳增长。地区生产总值在主动核减历史虚增数据、从严控制政府投资项目的情况下,预计增长6%;城乡常住居民人均可支配收入预计分别增长8%,快于经济增速;剔除虚增空转及营改增因素,一般公共预算收入完成70.5亿元,增长6.4%,其中,税收收入增长11.9%,非税收入占比降至22.5%,财政收入结构进一步优化。

一年来,我们主要做了以下工作:

一是认真整改中央巡视“回头看”和自治区党委巡视、中央环保督察和自治区生态环境保护大检查反馈问题。针对财政收入虚增空转和部分旗县工业增加值存在水分问题,主动核减虚增空转财政收入58亿元、占总量的45%,核减规模以上工业增加值464亿元、占规模以上工业增加值的67%。针对中央环保督察反馈问题,全部停退国家级自然保护区内的矿业权,建成运行全国同行业首例电解铝企业烟气污染物近零排放示范工程,关闭搬迁开发区煤炭交易市场。针对破坏草原和森林资源问题,制定了专项整改方案,对相关责任人进行了追责问责。针对政府过度举债问题,全面梳理政府投资项目,停建缓建政府投资项目40个、涉及金额87亿元。全面摸底排查全市债务情况,制定化解债务方案,积极稳妥开展化债工作,完成了年度债务化解任务。针对拖欠农民工工资问题,全面开展核查核实,政府投资项目拖欠的6亿元农民工工资全部足额拨付到位,按照“实名制”办法发放到农民工手中。

二是持续推进供给侧结构性改革。强化政策落实和“放管服”工作,推进“三去一降一补”任务落地见效。置换电解铝产能指标83.3万吨,取缔“地条钢”2.6万吨。与棚改工作相结合,消化房地产库存2.6万套。出台降本增效38条意见,落实“助保贷”、大用户直购电等综合措施,为企业减负20多亿元。

三是推动工业提质增效。以项目带动全链条发展,实施亿元以上延链补链项目160个,梅花生物苏氨酸、伯恩露笑蓝宝石二期等项目建成投产,金煤化工聚乙醇酸项目完成中试,邦杰食品肉牛加工项目试生产。34种主要工业品产量和价格大幅增长,规模以上工业企业利润增长8.4%。新能源、现代蒙医药、大数据等战略性新兴产业快速发展。促进企业创新平台建设,国家级高新技术企业达到26家,院士专家工作站达到7个。

四是加快农牧业现代化进程。建成农业精准节水面积152万亩,其中40万亩核心区实现水肥一体化和智能终端管理,主要农作物机械化水平达到85%。压减籽粒玉米248万亩,粮食总产稳定在百亿斤以上。坚持为养而种、种养结合,饲草料面积达到530万亩,粮经饲比例调整为68:13:19。加快肉牛育种改良,科尔沁肉牛种业基地投入运行。创新农牧业生产经营模式,紧密型农企利益联结比例达到55%。农产品“三品一标”认证产品新增43个、达到206个,认证面积达到300万亩。

五是培育发展现代服务业。组织实施“通辽文化旅游季”系列活动,科尔沁500公里风景大道列入自治区旅游品牌线路,13座营地、驿站运营态势良好,举办“8·18”哲里木赛马节、“亚洲巴哈·通辽一站到底”汽车越野拉力赛、全国公路自行车冠军赛等品牌赛事。健全金融监管服务体系,4个旗县农商行获批筹建。与农行、农发行、国开行等金融机构签署战略合作框架协议,实现融资85亿元。通霍铁路两次提速运行,内蒙古东部煤炭交易中心投入使用。开通大连港货运循环班列和集装箱冷藏班列,通辽丰盛农产品物流园建成运营,红干椒、肉牛等特色专业市场提档升级。电子商务网络零售额蒙东第一。养老服务、休闲健身等新兴业态快速成长。

六是全力推进双城同创。坚持“创建为民、创建惠民”,组织广大市民积极参与,同心同向、携手共建美好家园。深入开展“十大专项整治”行动,解决了一批群众关心、社会关切的老大难问题,中心城区拆墙透绿,拆除违法建筑,硬化背街小巷330万平方米,清运城乡垃圾110万立方米,攻克了“小广告”、占道经营、乱堆乱放等城市顽疾,城市面貌和文明程度明显提升。经过持续艰苦努力,成功创建国家卫生城市和全国文明城市。这一成绩来之不易,是全市各族干部群众用心血和汗水换来的荣誉!

七是加大精准脱贫力度。严格落实“六个精准”“五个一批”扶贫举措,4.6万贫困人口稳定脱贫。4个国贫旗列入京蒙帮扶计划。创新产业扶贫机制,通过土地流转、牲畜托养、入股合作等方式,带动4.14万贫困人口实现产业增收及就业。实施生态补偿脱贫1000人。对建档立卡贫困家庭中小学生和大学在校生给予定额补助。贫困人口免费体检8.86万人,覆盖率达到89%。实施“三个全兜底”政策,有效缓解了因病致贫问题。近万名符合条件的建档立卡贫困人口纳入低保。

八是加强生态环境保护。提交的《通辽市罕山国家级自然保护区条例》经市人大常委会表决通过并上报自治区待批,建立了市县乡三级河长体系。加快大气污染综合整治示范区建设,空气质量优良天数达到313天。对重点排污企业污染源实行在线、工况、视频“三位一体”24小时自动监控管理,取缔中心城区10蒸吨以下燃煤锅炉。现有污水处理厂主要污染物实现达标排放,启动了城市污水排放扩容提标工程。强化农业面源污染和投入品管控。完成科尔沁沙地“双千万亩”综合治理312万亩,实施了霍林河地区矿山生态修复治理工程。

九是着力保障和改善民生。千方百计增加民生投入,全市财政民生支出占总支出的78%。鼓励多渠道多形式就业,城镇新增就业1.8万人,实现零就业家庭动态清零。棚户区改造近6000套。科尔沁区、科左中旗、奈曼旗通过国家义务教育均衡发展达标验收。社会保障水平继续提高,退休人员养老金、城乡居民基础养老金月人均分别提高137元和20元,城乡居民最低生活保障标准月人均分别提高30元和25元。医疗保险实现异地就医直接结算。

十是维护社会大局和谐稳定。开展社会矛盾积案化解“百日攻坚”行动,有效解决重点群体信访问题。开展系列“警务技能大演练”,保持打击违法犯罪高压态势,现行命案全部侦破,成功侦破公安部督办超过15年特大疑难积压命案13起,破获公安部督办“9·05”特大网络赌博案。强化公共安全理念,全面落实安全生产责任制,加强食品药品市场监管,公共安全形势总体稳定。创新社会治理,推进政府职能法治化,市县乡三级政府法律顾问全覆盖,依法行政能力明显增强。

成绩来之不易,问题也不容忽视,突出表现在:欠发达的市情还没有根本改变,粗放型经济增长方式依然存在,转方式、调结构任务还十分艰巨;市场主体创新能力不强,民间投资活力不足,实体经济水平有待提高,固定资产投资增速放缓,缺少大项目、好项目支撑,稳增长的基础还不够牢固;主导产业层次不高,资源型产业链条短、附加值低,战略性新兴产业规模小,现代服务业发展不够充分;可用财力不足,防范化解政府债务风险的任务艰巨而繁重;生态环境仍很脆弱,经济发展与环境保护的矛盾依然突出;民生领域还有不少短板,城乡居民收入和基本公共服务低于全区水平,脱贫攻坚任务紧迫而艰巨,群众在就业、教育、医疗、居住、养老等方面还有不少难题;政府自身建设还需加强,依法行政能力和服务效能有待提高。我们要坚持问题导向,勇于担当,履职尽责,努力把政府工作做得更好。

二、今后五年总体目标和主要任务

今后五年,是我市决胜全面建成小康社会、开启现代化新征程的关键时期,我们要高举中国特色社会主义伟大旗帜,全面贯彻党的十九大精神,以习近平新时代中国特色社会主义思想为指导,持续深入落实习近平总书记考察内蒙古重要讲话和为内蒙古题词精神,坚持稳中求进工作总基调,坚持新发展理念,紧扣社会主要矛盾变化,按照高质量发展要求,统筹推进“五位一体”总体布局和协调推进“四个全面”战略布局,以供给侧结构性改革为主线,坚定实施“七大战略”,坚决打赢“三大攻坚战”,推动高质量发展,更好满足人民日益增长的美好生活需要。

贯彻上述指导思想,我们要始终把党的十九大精神贯穿政府工作全过程,按照“两个一百年、两个阶段”的战略安排,今后五年我市经济社会发展的主要目标是:

——综合经济实力实现新提升。以更高站位、更宽视野谋划发展,地区生产总值、固定资产投资、一般公共预算收入、城乡常住居民人均可支配收入增幅高于全区平均水平,综合实力和发展质量进一步提升,建设自治区发展的重要增长极。

——产业发展层次实现新提升。以全链条模式发展主导产业,构建实体经济、科技创新、现代金融、人力资源协同发展的现代产业体系,经济结构更加合理、核心竞争力明显增强,加快建设现代化经济体系。

——科技创新能力实现新提升。深入实施科教兴市和人才强市战略,关键技术攻关取得突破,高新技术企业、自治区级以上研发平台数量大幅增加,大众创业万众创新活力显著增强,科技对经济增长的贡献率稳步提高。

——改革开放水平实现新提升。重点领域和关键环节改革取得决定性成果,各方面体制机制更加健全完善,形成立足蒙东、融入东北,守望草原、面向渤海,东西集通、南北联开的对外开放新格局。

——生态文明建设实现新提升。生态保护修复重大工程深入实施,能源资源开发利用效率和绿色低碳水平大幅提高,主要污染物排放显著减少,生态文明制度更加健全,生态环境明显改善。

——居民幸福指数实现新提升。现行标准下农村牧区贫困人口全部脱贫,贫困旗县全部摘帽。就业更加充分,社会保障全覆盖,公共服务体系更加完善,均等化水平有新提高,民族团结进步事业蓬勃发展,“法治通辽”建设不断深入,各族群众获得感、幸福感、安全感显著增强。

实现上述目标,我们要谋在新处、干在实处、走在前列,努力把通辽建成充满创新创意活力、独具草原文化魅力、彰显富强民主文明和谐美丽的新时代草原名城。

今后五年的主要任务是:

(一)打造全产业链发展模式,推动资源地区转型发展。深入贯彻“五个结合”重要要求,坚持世界眼光、通辽优势,推动传统优势产业升级发展、战略新兴产业集聚发展、主导产业全链条发展,构建生态农牧业、玉米生物、有色新材料、科尔沁肉牛、现代蒙医药、煤化工、沙产业、会展业、文化旅游等多元发展的现代产业体系,建设资源地区转方式发展先行区。到2020年,主导产业链产值全部过百亿,其中玉米生物和有色新材料产业产值均过千亿。加快打造东北铝镍硅有色新材料产业基地、国家重要的玉米生物产业基地、中国草原肉牛之都、国际蒙医药之都、东北区域物流会展中心、一带一路“敖包相会”文化旅游名城。设立产业发展基金,发挥政府投资引导作用,激发民间投资活力。规范政府举债行为,强化金融监管,坚决打赢防范化解重大风险攻坚战。加大金融服务力度,支持实体经济发展壮大。

(二)实施乡村振兴战略,推进农牧业和农村牧区现代化。按照产业兴旺、生态宜居、乡风文明、治理有效、生活富裕的总要求,推进农牧业和农村牧区现代化走在自治区前列,建设农牧业和农村牧区现代化示范区。推动农村牧区基础设施建设提档升级,健全完善基本公共服务体系,重塑城乡关系,促进城乡融合发展。深化农牧业供给侧结构性改革,优化农牧业区域布局,大力发展生态节水农牧业,开展优势特色农畜产品标准化生产示范,打响“绿色科尔沁”品牌。深化以土地“三权”分置为重点的农村牧区综合改革,构建现代农牧业产业体系、生产体系、经营体系,发展多种形式适度规模经营,培育新型农牧业经营主体,推进农村牧区一二三产业融合发展。完善乡村治理体系,开展农村牧区人居环境整治,建设美丽宜居乡村。

(三)实施创新驱动发展战略,加快培育新动能。加强创新平台建设,培育高新技术企业和中小科技企业,在关键共性技术和成果转化等方面取得突破,建设主导产业创新创业集聚区。通辽经济技术开发区、霍林郭勒工业园区力争创成国家级高新技术开发区。实施“科尔沁英才”工程,开展高层次人才“万人培育引进行动”和科技人才引进培育“千人计划”。深化重点领域和关键环节改革,向改革要动力、要活力。大力发展非公有制经济,不断培育壮大市场主体。构建开放型经济新体制,建设融入国家“一带一路”和草原丝绸之路经济带重要节点城市。

(四)实施区域协调发展战略,构建城乡融合发展新格局。加快实施三大区域协同发展战略,推动北部霍扎一体化率先发展、中部两区一县一旗融合发展、南部三点一带跨越发展。落实“三个百万”人口规划布局,加快老城区改造提档、新城区高端提升、市政设施扩面提质,建设百万人口区域中心城市;全面提升城镇功能和产业支撑能力,承载吸纳百万人口就地就近城镇化;建设美丽乡村,发展现代农牧业,实现百万农牧民安居乐业。推进“三个市民化”进程,加快外来务工人员、农村牧区转移就业人口、城中村和城边村居民转为市民。规划建设一批基础设施互联互通工程,实施集通铁路电气化改造,推进通辽至乌兰浩特至齐齐哈尔高铁、鲁北至霍林河高速公路等项目前期工作,开工建设“LXB”供水通辽支线、“引绰济辽”二期等重大引水工程,加快“四好”农村公路建设。

(五)坚持绿色发展理念,全面加强生态文明建设。牢固树立和践行绿水青山就是金山银山的理念,统筹山水林田湖草系统治理,落实最严格的生态环境保护制度。大力发展绿色产业,打绿色牌,走生态路,建设环境友好型社会。持续开展大气、水、土壤污染防治行动,坚决打赢污染防治攻坚战。工业污染源全面达标排放,综合治理流域环境,促进废弃物无害化处理、资源化利用。实施千万亩农业高效节水、千万亩草原修复、千万亩天然草原现代畜牧业示范、千万亩科尔沁沙地治理四大生态修复保护工程,开展大规模国土绿化,建设北疆生态安全屏障功能区。

(六)激发文化创新创意活力,建设新时代草原名城。深入贯彻落实习近平总书记关于乌兰牧骑事业发展的重要指示精神,扶持乌兰牧骑创新发展,创作更多接地气、传得开、留得下的优秀作品,弘扬民族文化和时代精神,守好各民族共有的精神家园。

积极培育和践行社会主义核心价值观,深入开展群众性精神文明创建活动,加强青少年思想道德建设。实施文明城市创建提升工程,全面推进“八大环境”建设,持续巩固全国文明城市创建成果,努力建设崇德向善、和谐宜居、文化厚重的城市。完善文化管理体制和公共文化服务体系,推动公共文化资源向基层延伸,促进文化与旅游、体育、会展等产业融合发展,提高科尔沁文化软实力。

(七)发展民生社会事业,不断满足人民日益增长的美好生活需要。坚持尽力而为、量力而行,坚守底线、突出重点,完善制度、引导预期,让改革发展成果更多更公平地惠及全体人民。坚决打赢脱贫攻坚战,确保贫困人口和贫困地区同全国一道进入全面小康社会。实施积极的就业政策,促进高质量稳定就业。基本建成覆盖城乡居民的多层次社会保障体系。加快教育现代化,办好人民满意教育。推进健康通辽建设,提高全民健康水平。实施食品安全战略,提升食品药品安全保障水平。完善安全生产责任制,统筹推进安全生产领域改革,不断提升安全发展水平。实施军民融合发展战略,继续争创全国“双拥模范城”。扎实推进国防动员建设,全面提升国防动员潜力。加强和创新社会治理,建设高水平的“平安通辽”。深入开展民族团结进步教育和创建活动,建设新时代民族团结进步模范区。

各位代表,不忘初心,方得始终。只要我们保持永不懈怠的精神状态,登高望远、勇于创新、苦干实干,就一定能够开创现代化通辽的美好未来。

三、2018年工作安排

今年是贯彻党的十九大精神的开局之年,是改革开放40周年,是决胜全面建成小康社会、实施“十三五”规划承上启下的关键一年,政府工作要以习近平新时代中国特色社会主义思想为指导,深入贯彻中央经济工作会议精神,全面落实自治区党委十届五次全会暨全区经济工作会议和市委五届六次全会暨全市经济工作会议精神,坚持稳中求进工作总基调,坚持新发展理念,紧扣社会主要矛盾变化,按照高质量发展要求,加快建设现代化经济体系,以供给侧结构性改革为主线,推动质量变革、效率变革、动力变革,坚决打好防范化解重大风险、精准脱贫、污染防治攻坚战,守住发展、民生、生态三条底线,抓重点、补短板、强弱项,统筹推进稳增长、促改革、调结构、惠民生、防风险各项工作,促进经济社会持续健康发展。

主要预期目标是:地区生产总值增长6.5%左右;一般公共预算收入增长5%、完成74亿元,非税收入占比进一步下降;城乡常住居民人均可支配收入分别增长8%和9%左右;城镇新增就业1.7万人,城镇登记失业率控制在3.9%以内;单位地区生产总值能耗完成自治区下达的目标;供给侧结构性改革取得实质性进展;政府债务化解完成年度任务,财政金融风险有效防控;扶持3万贫困人口稳定脱贫;主要污染物排放总量大幅减少。这些目标体现了稳中求进的工作总基调,体现了高质量发展的根本要求,体现了以人民为中心的发展思想,体现了坚决打赢“三大攻坚战”的新时代要求。这些目标符合我市实际,经过努力是完全可以实现的。

决胜全面建成小康社会,我们必须打好“三大攻坚战”:

——打好防范化解重大风险攻坚战。化解政府债务是一项政治任务,按照自治区要求,进一步摸清政府债务底数,完善政府债务化解方案,压实化解责任,采取预算安排、压缩一般性支出、土地出让、盘活财政存量资金等措施,有计划、有步骤地开展债务化解工作。有效防控金融风险,坚决打击违法违规金融活动,整治非法集资、民间高利贷、恶意逃废金融债务等各类金融违法行为。切实加强地方法人金融机构风险防范,积极化解国有企业债务风险,加强金融风险预测预警,坚决守住不发生区域性系统性金融风险的底线。

——打好精准脱贫攻坚战。聚焦精准扶贫精准脱贫,动员全社会力量,构建大扶贫格局,落实京蒙对口扶贫,集中力量解决深度贫困问题。突出产业脱贫治本作用,激发贫困人口内生动力,引导贫困人口进入产业链增收。对有劳动能力的贫困人口精准落实脱贫产业;对没有劳动能力的贫困人口,尽可能通过土地流转、托管代养、发展光伏产业等方式,与企业建立利益联结机制,确保有分红。全面细化落实健康扶贫、教育扶贫措施,强化政策兜底保障,实现应保尽保。

——打好污染防治攻坚战。坚决抓好中央环保督察和自治区生态环境保护大检查反馈意见整改。深入实施大气、水、土壤污染防治行动计划,整治燃煤锅炉,北部地区电解铝企业烟气污染物近零排放示范工程稳定运行,提标改造污水处理厂,综合整治农业面源污染,加强土壤污染防治,着力解决突出环境问题。加强环境执法监管,发挥环境监管“三位一体”和环境保护“四级网格”监督作用,开展北部地区环境质量第三方监测,持续排查整治环境隐患,依法严惩环境违法行为,确保主要污染物排放稳定下降,生态环境质量稳步提高。

实现高质量发展,我们主要做好以下工作:

(一)以壮大实体经济为着力点,夯实稳增长基础。实体经济是通辽最现实的发展基础,坚持创新驱动,项目带动,不断增强发展质量优势。

深化供给侧结构性改革。坚持用市场化、法治化手段,在“破、立、降”上下功夫。大力破除无效供给,重组盘活停产半停产企业,把有限的水煤指标、土地资源、环境容量、金融信贷规模配置给优质企业。大力培育新动能,加快发展休闲健身、医养结合等新兴服务业态,推动互联网、大数据、人工智能和实体经济深度融合,在中高端消费、绿色低碳、共享经济等领域培育新增长点。加快建立多主体供给、多渠道保障、租购并举的住房保障制度,有效调整住房供给结构,增加中小户型和智能型、高品质住房供应,引导房地产企业进入农牧业、教育、医疗、旅游、养老等领域,促进房地产企业转型发展。大力降低实体经济成本,全面落实降本增效政策,继续清理涉企收费,加大对乱收费的查处和整治力度。积极推进大用户直购电交易,扩大“助保贷”覆盖面,降低物流成本和制度性交易成本。

激发各类市场主体活力。开展全产业链科技攻关,深化科技合作,加快组建玉米产业技术创新战略联盟、肉牛工程技术研究院,推动蒙医药国家级重点实验室等创新平台建设。制定出台非公有制经济发展政策措施,加大对非公有制经济的服务力度,完善市旗领导包联企业制度,支持非公有制经济发展壮大。全面实施并不断完善市场准入负面清单制度,推动大众创业万众创新,新增市场主体1.5万户以上。实施质量强市战略,开展质量提升行动,评选首届“市长质量奖”。深入实施企业和企业家培育工程,弘扬企业家精神,构建亲清新型政商关系,营造良好发展氛围,以软环境换取硬实力。

加强基础设施网络建设。毛都水库竣工蓄水,嘎海山水库主体完工,推进七泡子、乌兰哈达水库前期工作。通辽至京沈高铁新民北站铁路建成通车,完成京通铁路电气化改造。争取开工建设内蒙古省际大通道扎鲁特旗段高速公路、国道505线库伦至奈曼等3条二级公路,力争大沁他拉至白家湾子高速公路、国道111线舍伯吐至哈根庙一级公路、省道208线鲁北至开鲁二级公路建成通车。实施通辽机场改扩建工程,推进通辽至霍林郭勒天然气管道工程建设。实施农村牧区电网改造升级工程。

(二)加快转型升级,提高工业发展质量和效益。坚持发挥优势和补齐短板一起做,调整存量和做优增量同步抓,丰富上游、发展下游,推动主导产业高端化、智能化、绿色化发展。

促进传统产业迈向中高端。加快资源型产业延长链条,铝镍硅有色新材料产业向合金化、轻量化高端产品方向发展,力争福耀集团二期项目开工建设,联晟双零箔、伯恩露笑蓝宝石晶体三期等项目建成投产。玉米生物产业向食品级和医药级延伸,开工建设圣雪大成原料药、华北制药维生素C等项目,开封药业一期等项目建成投产。煤化工产业向新型精细化方向拓展,启动建设金煤化工聚乙醇酸项目,加快富德康乃尔重组步伐。沙产业向控、治、用融合发展,点沙成金、变沙为宝。

发展战略性新兴产业。促进大数据产业与工业大数据集成应用,建成运营中科曙光、九次方大数据平台,启动建设华为云计算中心。推动现代蒙医药产业向药食同源、康养一体发展,建设蒙医药康养中心,完成修正科尔沁药业蒙药基地、天奈药业项目主体工程。开工建设扎鲁特旗再生能源循环经济产业园项目,促进节能环保、清洁生产、清洁能源等绿色产业发展,培育一批具有创新能力的排头兵企业。

优化工业园区产业布局。按照全产业链发展要求,树立全市一盘棋思想,统筹规划,科学布局,实施园区振兴计划。以科尔沁工业园区、开鲁工业园区为重点发展玉米生物产业,以霍林郭勒工业园区、扎哈淖尔工业园区、奈曼工业园区为重点发展铝镍硅新材料产业,以扎鲁特高技术煤化工园区为重点发展现代煤化工产业,以科左后旗、科左中旗、扎鲁特旗、开鲁县为重点发展肉牛加工产业,以开发区、库伦旗、奈曼旗、科左后旗为重点发展现代蒙医药产业,以奈曼旗、科左后旗、库伦旗为重点发展沙产业。强化园区上下游企业间协同配套,探索铝水、淀粉乳直供销售,提高产业关联度。加强园区热电联产、中水回用等基础设施建设,提高工业园区承载能力,促进产业集群化发展。

(三)发展绿色生态农牧业,提升农牧业现代化水平。坚持质量兴农、绿色兴农,在节水农业、品牌农牧业、一二三产业融合发展上下功夫,加快农牧业由增产向提质转变。

持续推进农牧业供给侧结构性改革。发展生态节水农业,与土地规模经营、专业化集约化生产、水资源综合利用改革相结合,高标准建设节水高产高效粮食功能区400万亩。以市场为导向,发挥龙头企业和专业合作社带动作用,加快调整种植业结构,继续压减籽粒玉米种植面积,发展特色种植业340万亩。坚持为养而种、种养结合,建成550万亩优质饲草料基地。突出发展以肉牛为主的现代养殖业,实施肉牛提质扩繁工程,建立现代肉牛良种繁育推广体系,更新增加优质母牛20万头。

大力发展品牌农牧业。实施农牧业标准化战略,深入开展农畜产品质量安全县创建工作,建成优势特色农畜产品标准化生产示范基地39个,“三品一标”认证产品新增50个。加大耕地质量和土壤修复力度,推行清洁种养殖,实施化肥农药“双控”和无规定疫病区建设工程。完善以肉牛为重点的质量安全全程可追溯体系,建立主要农畜产品从农田牧场到餐桌的可追溯系统,打响科尔沁绿色农畜产品品牌。

促进农村牧区一二三产业融合发展。实施“互联网+现代农牧业”行动,拓展农牧业功能,延伸产业链条。大力发展产业化联合体,建立农牧业与金融、科技、流通等多业态融合发展的新机制,紧密型农企利益联结比例达到60%以上。规划建设农村牧区物流设施,健全旗乡村三级农畜产品和农资物流网络体系。建设中国草原肉牛之都,科尔沁牛业肉牛产业园、伊赛肉牛、邦杰食品等肉牛深加工项目投产达效,全市肉牛加工能力达到60万头。鼓励有条件的地方建设田园综合体,推动乡村新型产业发展,不断拓宽农牧民增收渠道。

(四)加快发展现代服务业,不断培育新的增长点。服务业是我们的发展短板,更是培育新动能的潜力所在,要运用新模式新业态推动现代服务业扩量提质。

促进文化旅游休闲产业融合发展。实施全域旅游、四季旅游、“旅游+”发展战略,进一步完善科尔沁500公里风景大道,突出文化系列主题,优化自驾车露营地、服务驿站、旅游小镇服务系统,完善旅游交通引导标识、交通干道与景区连接线配套功能。加快僧格林沁王府保护修复、哈民考古遗址、南宝遗址公园二期工程建设,提质升级山地草原、孝庄园等品牌景区,策划“一带一路”敖包相会文化旅游主题,办好“2018通辽文化旅游季”系列活动,打造“旅游+体育”品牌赛事,扩大文旅融合发展影响力。

促进商贸物流线上线下融合发展。完善中心城区长春欧亚、万达广场、万力城等核心商圈配套功能,加快大润发飞牛网、军利易购网上超市等商业智能化进程,建设一批配送中心和社区配送综合服务站,全力推进国家级电子商务进农村综合示范县项目建设。提升中心城区“五大物流平台”集聚功能,大力发展以甩挂运输、公铁海多式联运为重点的物流园区,加快旗县特色物流园区发展。建设中国(通辽)农产品电子交易中心、中国(通辽)肉牛电子交易中心、蒙中药材交易中心、内蒙古国际牛羊肉交易中心等现代化专业化市场,规划建设中心城区二手车交易市场。

促进金融业与实体经济融合发展。优化金融生态环境,发展绿色金融。培育引进各类金融机构,鼓励金融机构网点布局向县域延伸,争取内蒙古股权交易中心东部运营中心尽快落户。跟进落实与农发行等金融机构签署的战略合作协议,积极争取外国政府和国际金融组织贷款。深化金融体制改革,加快科尔沁区信用联社改制农商行步伐,深入推进县域金融工程改革试点,创建多层次资本市场,提高直接融资比重,拓宽融资渠道。

(五)推动重大改革举措落地,提升全方位开放水平。牢牢把握新时代全面深化改革“三个不能变”的要求,加快构建有利于高质量发展的体制机制。

加快重点领域和关键环节改革。深化“放管服”改革,对全部行政审批事项进行标准化梳理和流程再造,利用大数据、互联网等现代手段,实现审批服务事项“一窗受理”,优化办事服务,探索建立“最多跑一次”办事项目清单。深化电力体制和电力市场化改革,努力争取电力多边交易政策,以电价成本的新优势,打造招商引资和产业发展的投资“洼地”。推广区域微型电网试点经验,力促扎哈淖尔多能互补集成优化示范项目开工建设,构建局域智能电网新模式。深化国资国企改革,促进国有融资平台市场化转型,提高国有资本运营效率;建立完善现代企业制度,继续深化霍煤集团综合改革,完成通粮集团市场化改革。深化农村牧区综合改革,开展土地确权登记颁证,落实土地“三权”分置改革政策,开展土地承包经营权抵押贷款试点。深化农村牧区集体产权制度改革,壮大嘎查村集体经济,保障农牧民财产权益。推进农业水价综合改革,加快水务一体化改革。开展国有林场改革和供销社综合改革,创新优化“三农三牧”服务功能。深化科技体制改革,完善科技创新政策,鼓励市场主体建设科技创新平台,支持科技成果转化。深化社会体制改革,加快教育、文化、医药卫生体制和事业单位分类改革,充分释放社会领域发展潜力。

推动形成全面开放新格局。积极融入“一带一路”战略,主动参与和服务中蒙俄经济走廊建设。主动把开放前沿推进到发达地区、先进领域,顺应高铁时代,发展高铁经济,深度融入东北经济一体化发展,加强与京津冀、环渤海、长三角等发达地区合作。深化与清华大学、吉林大学、中科院以及区内市内院校合作,在关键技术攻关、基础教育、医疗卫生、人才交流等领域取得实质性成果。制定出台务实管用的招商引资和产业扶持政策,大力实施产业链精准招商,加快招商引资项目落地。培育会展经济,举办特色展会,启动建设国际会展中心。筹办国际肉牛产业大会、国际蒙医药博览会、科尔沁绿色食品博览会等有影响力的会展活动,让世界了解通辽,让通辽走向世界。

(六)坚决守住生态底线,持续用力改善生态环境。坚持人与自然和谐共生,尊重自然、顺应自然、保护自然,全面加强生态文明建设。

实施重大生态建设和修复工程。青山绿水、碧草蓝天是各族人民群众最大的福祉。从今年开始,全面实施“四个千万亩”生态建设和修复工程。实施千万亩农业高效节水工程,全面推行现代农业技术集成。实施千万亩草原修复工程,坚持自然修复为主,综合采取封育、禁牧、免耕补播飞播等措施,今年重点实施400万亩。实施千万亩天然草原现代畜牧业示范工程,坚持退牧还草、舍饲半舍饲、禁牧不禁养,划定草原生态红线,引导北部生态脆弱地区转变生产方式,建设现代化畜牧业示范区。实施千万亩科尔沁沙地治理工程,完成沙地治理630万亩,无立木林地造林100万亩。建设绿色矿山,推进退耕还林还草,加大水土流失综合治理力度。持续开展“三滥治理”,全面摸清森林资源底数,加大森林资源清理整治工作力度,坚决制止和惩处破坏生态环境行为,稳步提升生态系统质量,积极创建国家森林城市。

加强生态文明制度建设。全面落实主体功能区规划,加快生态保护红线、永久基本农田、城镇开发边界划定工作。严格执行《通辽市罕山国家级自然保护区条例》。落实最严格的水资源管理制度。调整林地保护利用规划。建立自然资源资产产权和用途管制制度,推行领导干部自然资源资产离任审计,建立生态环境损害赔偿和责任追究制度。

(七)巩固全国文明城市创建成果,建设宜居宜业美好家园。深入落实城市总体规划,完善市域城镇体系,强化民生城市建设管理,推动城市内涵式发展,提升城镇化质量。

加快完善中心城区城市功能。强化城市规划工作,编制重点区域城市设计。开展城市修补、生态修复工作。配合市人大常委会制定《通辽市城乡规划条例》,严格规划刚性约束。完善城市路网,加大征拆力度,推进福利路、新工一路、滨河大街、民主路立交桥和白音太来大街公铁立交桥引道及排水等工程,建设红光大街、新工四路、新工二路、东顺路高铁控制性节点工程,加快建设高铁站南广场,推动新世纪大桥两侧连接线及互通式立交工程、红光大桥等重点项目建设。高标准实施中心城区东出口环境综合改造工程。规划建设14处公共停车场、50座公共厕所。实施中心城区中水处理提标扩建工程,建设三义堂水源地,扩建通新净水厂。新建改造供热供水管网,实现新老城区互联互通。

加快建设特色镇和美丽乡村。全面提升城关镇产业支撑和服务功能,完善改造市政设施和公共服务设施,旗县城关镇公厕全部由旱厕改为水冲式厕所。集中规划建设融特色产业、文化旅游、社区服务等功能于一体的特色小镇,提高舍伯吐肉牛小镇、东风红干椒小镇建设水平。实施农村牧区人居环境整治三年行动计划,推进农村牧区户厕建设和改造,每个苏木乡镇建成1个生活垃圾污水治理示范村,农村生活垃圾治理率达到70%以上。保护传统村落和历史建筑,加强乡风文明建设,推进移风易俗,树立农村牧区社会新风尚。

持续提升城市文明程度。文明城创建只有起点没有终点。坚持以人为本,深入推进“八大环境”建设,加快构建政府与市场良性互动、人与自然和谐共生的城市自然与人文生态环境。大力实施意识形态统领、社会主义核心价值观铸魂、文明城市创建提升、文化惠民、科尔沁文化传承保护、文艺精品创作、文化产业培育七大工程,提升城市文明程度和文化品位。

提高城镇精细化管理水平。认真执行《通辽市城市市容和环境卫生管理条例》,依法对难管理、易反复的城市顽疾进行常态化、规范化整治,促进城市环境提档升级。加快推进城市管理体制综合执法改革,理顺体制,完善机制,加强力量,落实责任,实现科学管理、精细服务。配合市人大常委会制定物业管理条例,加强弃管楼、老旧小区物业管理,用“绣花功夫”管理城市。加快建设“智慧通辽”,编制智慧城市总体方案,一次规划、分步实施,整合资源、共建共享,消除“信息孤岛”,实现城市管理服务现代化、智能化。

(八)大力发展社会事业,努力增进民生福祉。坚守民生底线,办好民生实事,让群众有更多获得感。

全力推进保障体系社会化。在财力可支撑的基础上,解决好民生突出问题。全面解决就业结构性矛盾,完善创业扶持政策,实现比较充分和高质量就业。提高事业单位公开招聘效率,畅通人才引进绿色通道,满足用人需求。构建和谐劳动关系,维护农民工合法权益。实施全民参保计划,持续扩大社会保险覆盖面。统筹城乡社会救助体系,完善最低生活保障制度,提高城乡低保人均补助水平。完成棚户区改造1.7万套,建成公共租赁住房500套,继续实施老旧小区改造。积极应对人口老龄化,构建养老、孝老、敬老的政策体系和社会环境,建成使用市老年人养护中心,新建标准化医养结合养老机构10所、示范性社区居家养老服务中心20家,大力改善农村牧区敬老院条件。

全力推进社会事业现代化。优先发展教育事业,全面提高教育质量。加强教师队伍建设,加大教师招录补充工作力度。规范办学行为,提高办学质量,着力解决中小学生课外负担重、“择校热”、“大班额”等突出问题。加快农村义务教育薄弱学校改造,推进义务教育均衡发展,确保开鲁县、科左后旗通过国家评估认定。大力发展普惠性幼儿园。规划建设内蒙古民族大学新校区,开工建设通辽一中教学综合楼,推进通辽蒙中改扩建工程,工业职业学校开学启用。加强医疗卫生服务体系和全科医生队伍建设,推进建设市医院新院区,继续创建“十强卫生院”“百佳卫生室”,采取以奖代补方式支持中心卫生院建设。完善医疗急救区域网,新建急救分站10个,开工建设通辽市中心血站。加强医疗卫生对外交流协作,落实京通、吉通卫生合作项目,抓好重点学科建设。深入开展爱国卫生运动,加强传染病、地方病等重大疾病和职业病危害防治。完善蒙医药服务网络,加快蒙医药事业发展。促进生育政策和相关经济社会政策配套衔接,提升妇幼保健水平,建设市妇幼保健院。启动创建国家食品安全示范城市,完善科尔沁绿色食品加工园区配套功能,继续推进“五小一场”升级改造。加强公共文化服务体系建设,扩大本地广播电视节目覆盖面。启动非遗传承保护和文化精品创作扶持奖励工程,建设非物质文化遗产数据库。广泛开展全民健身活动,加大公共体育设施投入,加快建设新城区全民健身中心和自治区东部青少年足球训练基地。发展民族团结进步事业,依法管理宗教事务,争创全国民族团结进步创建活动示范市。深入实施“七五”普法规划,落实国家机关“谁执法谁普法”责任制,加强公共法律服务体系建设。做好新形势下全民国防教育、人民防空和双拥优抚安置工作。加强困境儿童、留守儿童关爱保护。支持工会、共青团、妇联等人民团体和社会组织发挥作用,加强外事、侨务、档案、史志、气象等工作。

维护社会和谐稳定。树立大安全观,确保公共安全。加强信息化、立体化社会治安防控体系建设,严密防范和坚决打击危害国家安全和社会稳定的违法犯罪活动,开展积案破案攻坚,推动社会治安状况持续好转。完善“大信访、大维稳”工作格局,抓好信访维稳工作。实施“雪亮工程”,健全完善社会矛盾纠纷排查化解工作机制,加强预防和化解社会矛盾。弘扬生命至上、安全第一的思想,完善安全生产责任制,加强重点行业、重点领域安全专项整治,坚决杜绝重特大安全事故发生。

四、加强政府自身建设

全市各级政府及工作人员必须把政治建设放在首位,牢固树立“四个意识”,坚定“四个自信”,以坚强的政治保障提高公信力和执行力,努力建设人民满意的服务型政府。

坚持党的领导。党政军民学,东西南北中,党是领导一切的。要旗帜鲜明讲政治,坚决贯彻党中央决策部署,始终在政治立场、政治原则、政治道路上同党中央保持高度一致。把学习十九大精神作为当前和今后一个时期的头等大事和首要政治任务,在学懂、弄通、做实上下功夫,切实把思想和行动统一到十九大精神上来,用习近平新时代中国特色社会主义思想武装头脑、指导实践、推动工作。学习弘扬“红船精神”,在苦干实干中走在时代前列。认真开展“不忘初心、牢记使命”主题教育,在落实全面从严治党要求上做表率。

推进依法行政。牢固树立法治信仰和法治思维,政府工作人员要尊法、守法、学法、用法,全面提高依法行政能力。严格按照法定权限和程序履行职责,强化行政权力运行制约和监督,让政府工作在法治轨道上运行。健全依法决策机制,成立法律咨询专家委员会和重大行政决策咨询论证专家库,落实行政决策法定程序。健全行政执法人员管理制度,严格规范公正文明执法。持续加大“放管服”力度,减少行政审批事项、优化行政审批流程,提高精准服务能力。

增强政府诚信。政府诚信是社会诚信的风向标,各级政府及工作人员要加强诚信建设,市委、市政府作出的决策要勇于担当、必须担当,负责任地兑现承诺,坚决做到言必行、行必果。政贵有恒,要保持各项政策措施的连续性、稳定性,坚持“一张蓝图绘到底”、一茬接着一茬干,坚决做到新官也要理旧账。牢固树立正确政绩观,坚持实事求是,说实话、摸实情、出实招、办实事,以实实在在的发展成效赢取各界信任支持。建立健全守信激励、失信惩戒机制,以政府诚信引领社会诚信。

坚持实干担当。政府是“作战部”,不是“清谈馆”,对市委确定的目标、议定的工作、出台的文件,要坚持马上就办的工作作风,坚决做到有部署、有落实、有督查、有反馈、有问责,确保各项工作部署落到实处、见到实效。健全责任落实体系,对重点工作、重点项目实行专人专项推进,政府及工作部门主要负责同志要勇于挑最重的担子、敢于啃最硬的骨头、善于攻最强的堡垒,不达目的不罢休。要加强学习和实践,培养专业能力,弘扬专业精神,大兴调查研究之风,找准短板弱项,解决实际问题,增强推动高质量发展的实干本领。

保持清正廉洁。严格落实全面从严治党责任,切实抓好政府系统党风廉政建设。严格执行中央八项规定精神和自治区党委、市委配套规定,坚决纠正“四风”问题。加大审计监督力度,推进审计全覆盖。坚决整治不作为、慢作为、乱作为,让公务人员知敬畏、存戒惧、守底线。建立健全激励机制和容错纠错机制,为干事创业者营造放心做事、放手做事、放胆做事的工作环境,让干净干事、务实奉献成为全市上下的自觉行动和共同追求。

各位代表,使命呼唤担当,使命引领未来。让我们更加紧密地团结在以习近平同志为核心的党中央周围,在自治区党委、政府的正确领导下,在市委的坚强领导下,主动接受人大法律监督、政协民主监督和社会舆论监督,与全市人民想在一起,干在一起,为决胜全面建成小康社会、谱写“建设亮丽内蒙古、共圆伟大中国梦”的通辽新篇章而努力奋斗。
